# Highly efficient piezocatalytic composite with chitosan biopolymeric membranes and bismuth ferrite nanoparticles for dye decomposition and pathogenic *S. aureus* bacteria killing

**DOI:** 10.3389/fchem.2024.1420040

**Published:** 2024-06-06

**Authors:** Yunhong Liu, Jhilik Roy, Shubham Roy, Nur Amin Hoque, Bing Guo

**Affiliations:** ^1^ Department of Clinical Lab, The People’s Hospital of Longhua, Shenzhen, China; ^2^ Department of Physics, Jadavpur University, Kolkata, India; ^3^ School of Science, Harbin Institute of Technology, Shenzhen, China; ^4^ SAIS Department, Indian Association for the Cultivation of Science, Kolkata, India

**Keywords:** composites, piezocatalysis, chitosan doped BFO, dye degradation, nanoparticles

## Abstract

Untreated wastewater harbors dangerous pathogens, chemicals, and pollutants, posing grave public health threats. Nowadays, there is a rising demand for eco-friendly technologies for wastewater treatment. Recently, piezoelectric materials-based wastewater treatment technology has captured considerable interest among researchers because of its noninvasiveness and rapidity. Herein, a highly efficient piezoelectric composite material is designed with chitosan-incorporated bismuth ferrite (BFO) nanocrystals, to decompose pollutants and ablate bacteria in wastewater. On one hand, piezoelectric BFO has shown exclusive piezo-coefficient for ultrasound-mediated reactive oxygen species (ROS) production. On the other hand, chitosan depicts its biocompatible nature, which not only promotes cellular adhesion but also significantly elevates the ROS production capabilities of BFO under ultrasound. The synergistic effect of these two piezoelectric units in one composite entity shows an improved ROS production, eradicating ∼87.8% of Rhodamine B within 80 min under soft ultrasound treatment (rate constant, k ≈ 0.02866 min^−1^). After performing the scavenger experiment, it has been found that hydroxyl radicals are the dominating factor in this case. Further, the reusability of the composite piezocatalyst is confirmed through multiple cycles (five times) of the same experiment. The high polarizability of the composite material facilitates the generation of piezoelectric power through finger tapping (∼12.05 V), producing substantial instantaneous piezo-voltage. Moreover, the sample exhibits remarkable antibacterial activity, with nearly 99% bacterial eradication within 30 min. This indicates a significant advancement in utilizing biopolymeric composites incorporated with BFO for fabricating versatile devices with multidimensional applications.

## Graphical Abstract|

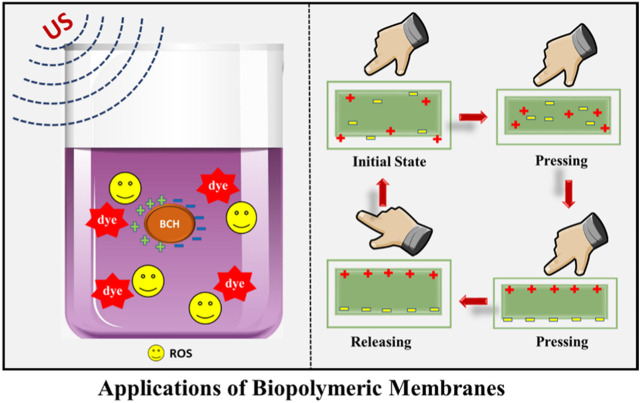

## 1 Introduction

Water pollution is caused by inadequate, agricultural overflow, sewage treatment, pharmaceutical waste, industrial discharge, and organic dyes, which threaten human health and aquatic biota. (Copaciu et al., 2013; Ismail et al., 2019). There is an urgent need to combat water pollution for both people and the environment. Globally, the textile industry releases nearly 40,000–50,000 tons of dyes regularly, which reduces oxygen in water bodies. (Curteza, 2020). Dyes that degrade into carcinogenic complexes contribute to various types of skin infections and cancer. (Ghaly et al., 2014). These sources of pollution must be addressed to protect human health and maintain aquatic biodiversity. Different methods have been adapted for degrading dye molecules such as coagulation, Fenton oxidation, adsorption, photocatalysis, and electrochemical methods. (Roy, 2021). However, all the methods have their limitations. Firstly, the adsorption-based method generally utilized to remove dye due to its efficacy, transfers adsorbents to a new phase, consequently leading to secondary pollution. (Senguttuvan and Senthilkumar, 2021; Uddin and Ampiaw, 2021). Fenton oxidation, though potent, functions within a fine pH range and presents challenges in control. Bioremediation, while economical and eco-friendly, may come across some issues such as reversibility and prolonged reaction times if not carefully managed. (Ramos and Santana, 2021). The coagulation method is advantageous in water discoloration by dye removal without decomposition but still, it has a shortcoming in removing very low molecular weight and the disposal of coagulant residuals. (Gadekar and Ahammed, 2016). Conversely, the photocatalysis process stands as a prevalent method compared to other alternatives. Nonetheless, achieving effective charge separation in photocatalytic materials remains challenging, and catalysts may corrode under low pH conditions. (Schneider et al., 2014; Ameta and Ameta, 2016). Hence, it is imperative to employ a viable approach to address these issues, which can effectively contribute to wastewater treatment by efficiently degrading dyes.

The piezocatalysis method is treated with piezo-electric materials having a non-centrosymmetric structure which is capable of converting mechanical energy into electrical energy. (Feng et al., 2017). Due to mechanical force, these materials undergo physical distortion, inducing charge center separation which causes polarization within the material. (Tu et al., 2020). This polarization, in turn, generates reactive oxygen species (ROS), initiating the catalytic process. (Chen et al., 2019). Furthermore, this approach exhibits superiority compared to the previously discussed techniques such as photocatalysis, adsorption, electrocatalysis, etc.

Piezo-responsive materials are increasingly important for their dual capabilities in wastewater treatment and energy harvesting (Table 1). (Mondal et al., 2022a) In piezoelectric effect, materials convert mechanical energy into electrical energy. It is recognized as a green technology. (Mondal et al., 2022b). So far, the developed piezo-responsive materials in the literature mostly include natural/synthetic organic polymers (e.g., PVDF, PLA, chitosan, and PLLA), and synthetic inorganic materials (e.g., barium titanate, MoS_2_, and bismuth ferrite (BFO)). Most recently, organic polymers have received increasing attention in piezoelectric applications such as chitosan, and PLLA, because of their appealing good biocompatibility, degradation capability, and even certain antibacterial activity, *in vitro* and *in vivo*. However, organic polymers have rarely been used in the field of piezocatalysis, which is mainly due to their relatively low piezoelectric coefficients. Thus, there is a pressing need to formulate highly efficient piezoelectric polymers with enhanced performance in dye decomposition and bacterial killing, although it is challenging.

**TABLE 1:** A comparison between different piezocatalysts and their catalytic efficiencies for dye degradation and bacterial mortality.

Name of the composite material	Degradation %	Degradation time (min)	References
TiO_2_/PVDF	Methylene blue (100%)	40	Dong et al. (2017)
Ag loaded LiNbO_3_/PVDF	Tetracycline (69%) ciprofloxacin (53%), *E. coli* (99.999%) and *S. aureus* (96.65%)	120(TC, CF); 180(EC, SA)	Singh et al. (2021)
ZnO/PVDF, ZnO/PDMS	Rhodamine B (RhB) (∼35%) for PDMS (∼90%) for PVDF	100	(Wu et al., 2020)
Barium strontium titanate-PDMS composite	Rhodamine B (97.8%)	90	Qian et al. (2020)
PDMS/MoS_2_	Rhodamine B (67%)	200	Lin et al. (2017)
PDMS/WS_2_ nanoflower	Rhodamine B (99%) *E. coli* (99.99%)	90	Masimukku et al. (2018)
Chitosan modified montmorillonite	Acid orange 7 (98%), and basic red (82.74%)	60	Karaca et al. (2017)
**BCH composite composed of chitosan-doped bismuth ferrite**	**Rhodamine B (87.8%) *S. aureus* bacteria (99%)**	**80, 30**	**This work**

Composite technology with a rational combination of different components has been widely used in material formulation to yield boosted output than the single component alone (Ma et al., 2022; Sarkar et al., 2023). In this contribution, we selected chitosan biopolymer membrane and BFO NPs to formulate a piezoelectric composite for dye decomposition and bacterial killing. For chitosan biopolymer membrane, exhibits natural biocompatibility with good cellular adhesion, antibacterial effect, and moderate piezoelectric voltage (12.05 V) and shows cases in the application of biomedical devices, drug delivery carriers, and implant coating. After doping bismuth ferrite (BFO) NPs into the chitosan biopolymer, the piezoelectric property of the composite would increase significantly due to the screen charge effect mediated e-h separation and charge carrier migration (Roy et al., 2022; Roy et al., 2024). The study examines the functionality of this polymer nanocomposite material, showcasing its dual capabilities as a piezoelectric energy harvester and a piezocatalyst. This illustrates its promise in generating sustainable energy and facilitating wastewater treatment.

**FIGURE 1:**
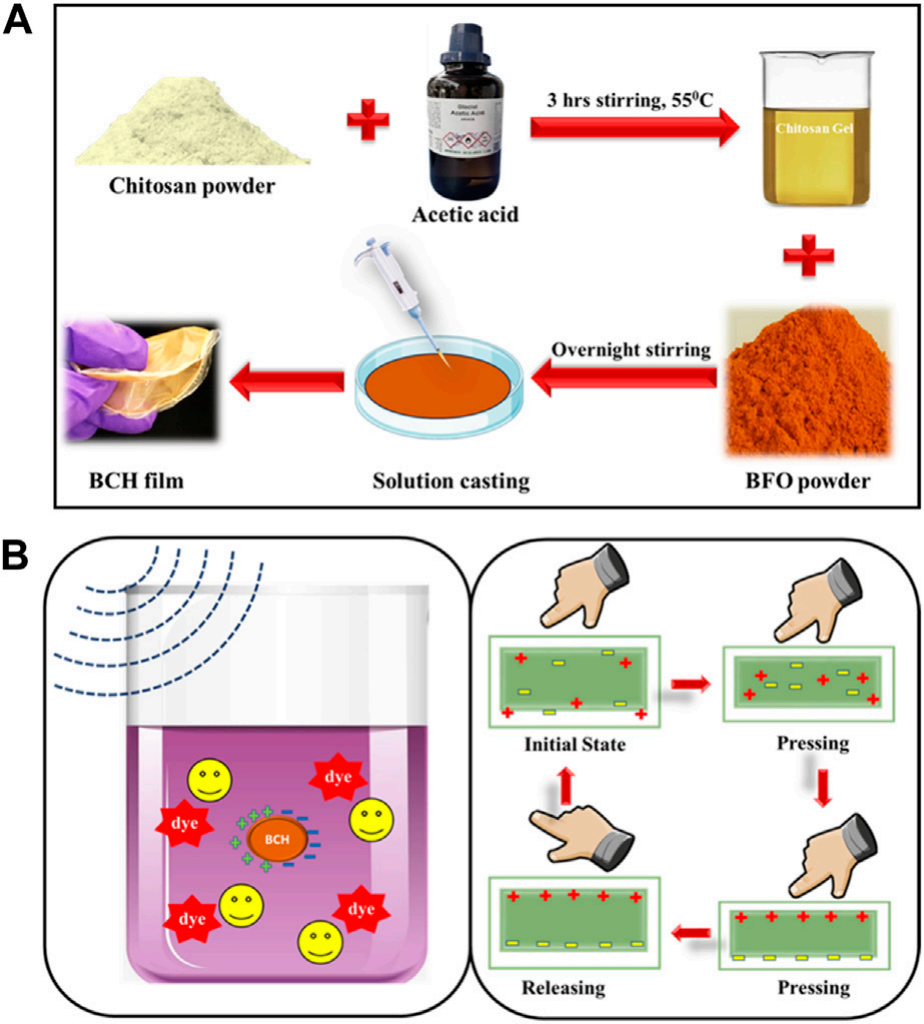
**(A)** Synthesis method of BCH composite with BFO doped chitosan nanocomposite; **(B)** applications of the biopolymeric nanocomposite membrane.

The BCH composite with bismuth ferrite NPs incorporated chitosan was synthesized in four different doping percentages, such as 0%, 5%, 7.5%, and 10%, and marked as BCH0, BCH5, BCH7.5, and BCH10 Figure 1. All of these nanocomposite membranes were characterized using different characterization tools to obtain their structural (XRD, FTIR), morphological (FESEM, TEM), electrical, and other physicochemical properties. Furthermore, the electrical properties were investigated to determine the extent of piezoelectric polarization reached in this nanocatalyst, establishing its potential as a promising piezoelectric material. Moreover, the composite material (BCH 7.5) shows the highest piezoelectric effect which is further used to degrade the Rhodamine B dye efficiently, using ultrasonic stimulation having an energy of 15 kHz. The degradation rates were found to be quite high and rapid (∼87.8% for RhB in 80 min) along with pathogenic *S. aureus* bacteria efficiently (99% in 30 min) by using a simple ultrasonic stimulation (15 kHz). The reusability of the catalyst further confirmed the outstanding stability and can be used repetitively more than five times in a row. We expect this research to show a transition in this innovative technology in practical applications.

## 2 Results and discussion

Herein, we designed a new piezoelectric nanocomposite (BCH) by using BFO to dope chitosan membrane via a facile two-stage solvothermal and solution casting method, which would perform highly efficient piezocatalytic ROS-mediated degradation of Rh-B and pathogenic *S. aureus* bacteria under soft ultrasound stimulus. The synergistic piezoelectric effect is derived from the band edge tilting and screening charge effect of the prepared nanocomposite. The as-synthesized samples were initially characterized by using several structural and morphological characterizations, followed by their piezoelectric ability to produce ROS for catalytic applications. This section deals with such results with their plausible rationale for a conclusive remark.

### 2.1 Structural, morphological, and stability analyses of the nanocomposites

**FIGURE 2:**
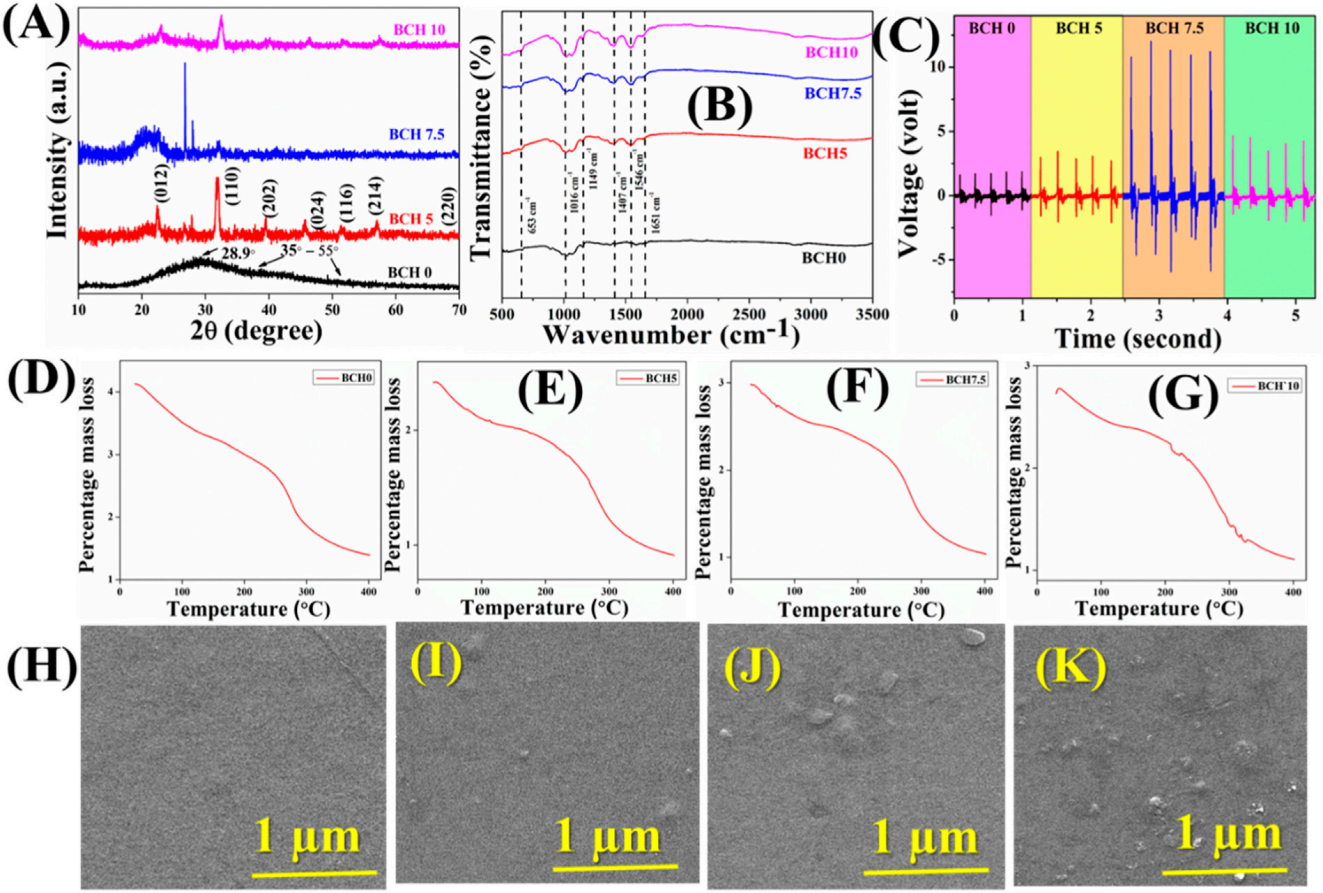
**(A)** X-ray diffractogram of BCH samples **(B)** FTIR spectra of the synthesized BCH sample; **(C)** Shows the time vs voltage graph for different BCH samples; TGA analysis of **(D)** BCH 0 **(E)** BCH5 **(F)** BCH 7.5 **(G)** BCH10; The FESEM micrographs of **(H)** BCH 0 **(I)** BCH5 **(J)** BCH 7.5 **(K)** BCH10.

Initially, the XRD of the nanocomposites was obtained to investigate their purity and other physicochemical properties. Figure 2 illustrates the X-ray diffraction (XRD) pattern of the pristine chitosan (CS) film (BCH 0), 5% doped BFO into chitosan matrix (BCH 5), 7.5% doped BFO into chitosan matrix (BCH 7.5), 10% doped BFO into chitosan matrix (BCH 10). Notably, the pure CS membrane exhibits a prominent crystalline peak at 2θ = 28.9°, corresponding to the reflection planes of (220). (Aziz et al., 2017). Earlier investigations have shown that the rigid crystalline structure of chitosan primarily arises from intramolecular and intermolecular hydrogen bonds. These bonds play a pivotal role in establishing the average intermolecular spacing within the crystalline sections of chitosan. The presence of a wide peak nearly at 35°–55° corresponds to the amorphous region of CS. (Belamie et al., 1999). After the incorporation of BFO into the CS matrix, several characteristic peaks of BFO have arisen and increased with the increment of doping percentages. (Rani et al., 2022). These peaks further confirm the successful synthesis of BFO NPs into the chitosan matrix.

The bonding networks of the fabricated membranes (BCH) were investigated by FTIR in the region of 500–4,000 cm^−1^ and depicted in Figure 2. (Roy et al., 2019). The FTIR bands of the pure bismuth ferrite NPs are depicted in the supplementary section (Supplementary Figure S1). (Biasotto and Simões, 2011; Gokul et al., 2021) The intense vibrational bands correspond to chitosan biopolymer at 1,016 as a result of the C-O stretching. The vibrational band at 1,149 cm^−1^ is for the C-O-C asymmetric vibrations. Whereas the bands at 1,407 cm^−1^ are ascribed to the -CH_2_ bending and vibrations (Magee, 2024). The vibrational peak at 653 cm^−1^ is due to the Fe-O stretching and 1,651 is for C=O stretching for amide-I. Besides these vibrational bands of chitosan, intense vibrations occurred at 1,546 cm^−1^ in all the doped samples which are increasing with the increasing percentage of BFO nanoparticles into the biopolymeric matrix (Fernandes Queiroz et al., 2015). This peak is due to the N-H bending of amide-II. This type of increment in the vibrational band suggests the successful incorporation of BFO NPs in the biopolymeric matrix (Magee, 2024). In summary, all the conventional characterization techniques suggest the successful fabrication of NPs incorporated bio-polymeric nanocomposite membranes.

Field Emission Scanning Electron Microscope (FESEM) was used to investigate the microstructure and the morphological analyses of the nanocomposite films. (Govindan et al., 2012). Figure 2H shows the FESEM images for pure and composite membranes. The surface of the film appears notably smooth for BCH0, whereas significant surface roughness can be seen for BCH5, BCH 7.5, and BCH 10 respectively. These micron-sized regions are homogenously dispersed into the chitosan surface and are increased with increasing the BFO doping percentages. These changes confirm the successful synthesis of BFO NPs into the chitosan film. In order to validate the morphology of the BFO nanoparticles, the Transmission Electron Microscopy (TEM) technique has been adopted herein (Supplementary Figure S2). Supplementary Figure S2 depicts an irregularly clustered, randomly distributed pattern of bismuth ferrite NPs. The mean diameter is around ∼35–40 nm, which is analogous to the FESEM images and provides a sufficient validation of the surface roughness of the composite membranes. From these morphological analyses of the composites, it has been verified the fact that the incorporation of BFO has been successful inside the chitosan biopolymeric matrix. Moreover, the increment of the surface roughness found in Figures 2I–K validates that BFO nanoparticles have been incorporated into chitosan.

Figure 2 also depicts the TGA curves for both the pure and doped chitosan films. According to the TGA curves, two notable weight loss stages were observed. The initial slight decrease in weight occurred between 50°C and 140°C, likely attributed to moisture vaporization. Subsequently, a second weight-loss phase was seen between 250°C and 350°C, indicative of the thermal degradation of the films. The loss rates were approximately ∼10% during each of these weight loss stages. Such a small weight loss indicates the temperature stability of the composites. In reality, ultrasound initiates a rapid increment in the temperature evolution in the catalysts. Such a sudden increment may decompose the structure/property of the sample. However, in our case, the insignificant weight loss validates the thermal stability of the synthesized nanocomposites.

### 2.2 Estimation of the piezoelectric properties of the samples

Understanding the process of producing piezoelectric stimulation triggered by external mechanical stimulus involves examining the dipolar polarization of the BCH sample. Researchers have reported that the motion of electrical dipoles inside the material can be affected by the frequency of external stimulation. (Yu et al., 2022). Application of external pressure generates dipolar polarization in the sample, causing a separation of positive charges which travel towards the negative one. A potential difference is created between the two electrodes as a result of this charge separation. Due to the external force, through an external circuit, the charge carriers travel in the opposite direction towards the electrodes, thereby creating a counteracting potential drop. (Mhatre et al., 2015).

Furthermore, to measure the electric voltage of the sample, a finger-tapping experiment was directed. To create the devices, the surface area of the nanocomposite membrane was 1 cm^2^ (Figure 2C). Aluminum electrodes were employed on both sides of the membrane. and with the help of copper wires, the connections were assembled. The electrical characteristics of the sample were consequently examined. The calculation of the force is depicted in the supplementary section (S3). For BCH 0, BCH 5, and BCH 10, the voltages are 1.88 V, 3.52 V, and 4.71 V respectively. The voltage for BCH 7.5 is the highest (approximately 12.05 V) compared to others. This increase clarifies why BFO NPs are present in the chitosan matrix, which enhances the polarization of the catalyst even more (Figure 2). Enhanced polarizability due to heightened doping percentages improves the performance of the membrane. However, higher doping concentrations hinder rapid dipole response to changes, leading to a decline in voltage over time (Ghosh et al., 2023). This phenomenon is ascribed to the influence of Maxwell-Wagner polarization (Prodromakis, 2009).

### 2.3 Piezocatalytic degradation of RhB by the nanocomposite

To assess the piezocatalytic effectiveness of the catalyst, BCH 7.5 membrane was used as it has shown the highest piezo effectiveness. The study employed the carcinogenic Rhodamine B (RhB) dye for experimentation. In the present study, the findings reveal that the degradation percentage of RhB dye is nearly 87.8% for BCH 7.5. Additionally, the BCH sample exhibits a rate constant (k) of 0.02866 × 10^−2 ^min^−1^ for the dye, further augmenting polarizability and facilitating RhB degradation. It was observed that the original dark pink color of RhB dye gradually changed to almost transparent after 80 min of ultrasonic treatment (Figure 4(B inset)) whereas for the control samples, the degradation rate is nearly 32% which is very nominal.

Utilizing BCH7.5 film for water purification offers significant advantages, such as reusability, attributed to the inherent mechanical and piezocatalytic robustness of the NPs. To calculate the robustness of the sample, a reusability test was done using the same film and a fresh solution of Rhodamine B (20 mL, 2.5 ppm). The results exhibit consistent catalytic efficacy over five cycles, maintaining efficiency levels nearly the same as the first cycle (Supplementary Section S7). This demonstrates the exceptional resilience and long-term durability of the chitosan-based biopolymeric sample.

Furthermore, water quality assessment data was obtained using a HANNA-19B portable pH/EC/TDS/Temperature meter and is presented in Table 2 both before and after the experiment. Total Dissolved Solid (TDS) and conductivity values of water samples the water quality. After incorporating organic dye and catalysts, both the TDS and conductivity values increased, subsequently declining after the catalytic experiment. In wastewater treatment applications, the post-catalysis results closely match the initial data, indicating that the piezocatalysts do not alter water quality and contribute to increased purity. Hence, it can be said that the as-synthesized sample not only reduces the load of the organic dye (Rh-B) but is also capable of retaining all the initial physical properties of the treated water sample.

**TABLE 2:** Water quality assessment test before and after the catalysis.

Parameters	pH	TDS (ppm)	Conductivity (μS/cm)
Before catalysis	6.6	2.1	4.2
Control (only dye)	6.7	6.2	10.0
Sample + dye	6.7	9.2	17.8
Catalysis (Before extraction of the NPs)	7.8	4.1	14.0
Catalysis (After extraction of the NPs)	6.7	3.2	8.5

#### 2.3.1 The fundamental mechanism behind piezocatalytic dye degradation

**FIGURE 3:**
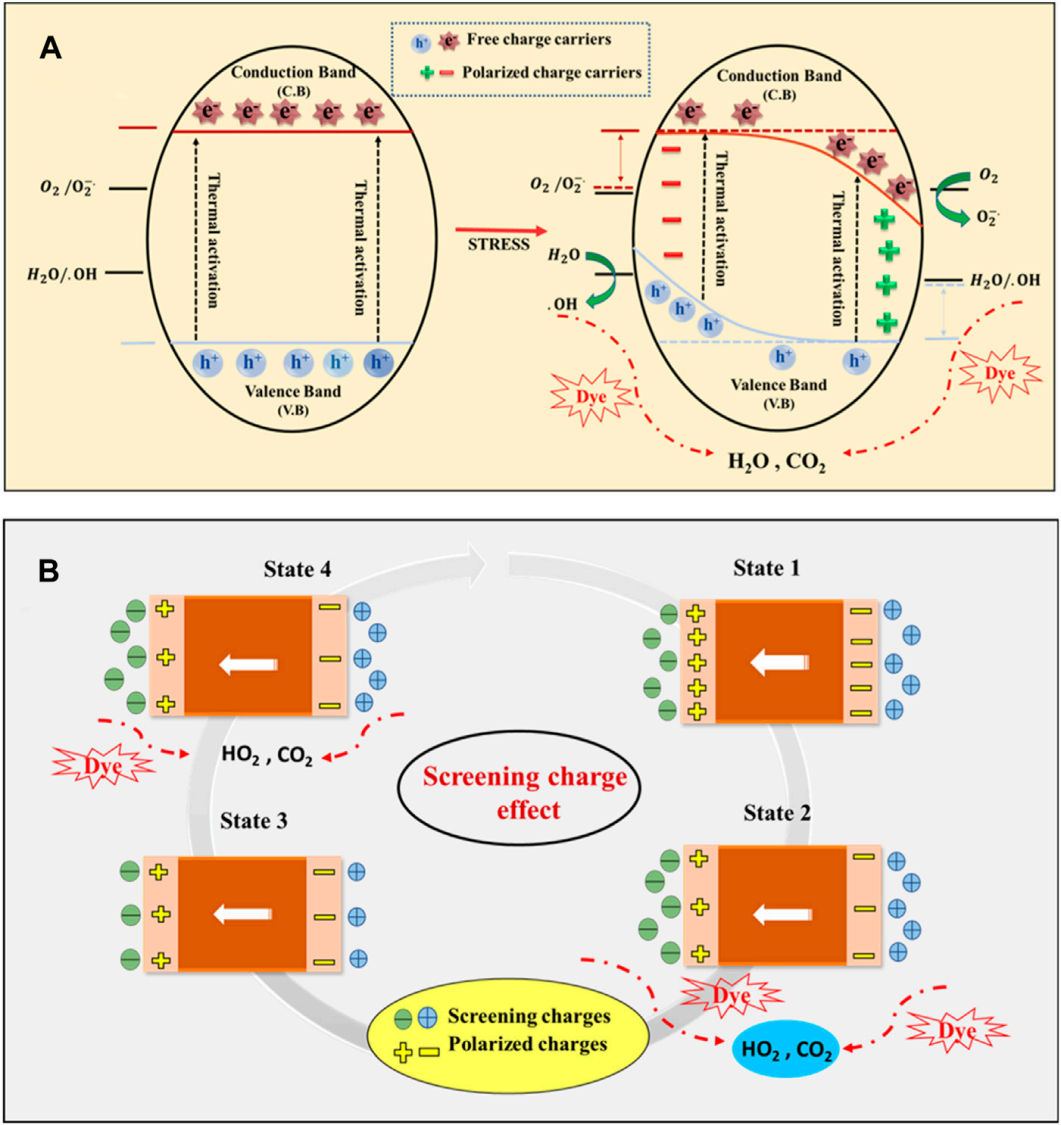
Underlying mechanism of piezocatalysis method **(A)** Energy band theory **(B)** Screening charge effect.

The piezocatalytic degradation utilizing the BCH composite has a specific mechanism (Figure 3). An internal electric field is created in the piezoelectric material when external pressure is applied. effectively segregating and sustaining the charge carriers. Dye degradation occurs through the generation of Reactive Oxygen Species (ROS). These ROS interrelate with the water molecules. ROS (•O_2_-/•OH) are produced when an interaction occurs between the charge carriers on the surface of the nanocomposite and the ions from the water molecules. This interaction sets off a redox reaction and polarization, which in turn starts the destruction of dye molecules. These radicals can captivate in reactions through two pathways: 1) the Screening Charge Effect and 2) the Energy Band Theory.

The BCH piezo catalyst initially maintains an equilibrium state (State 1) when immersed in the electrolyte-containing dye, complying with its ingrained polarization with screening charges. While subjected to external pressure, the movement of charge carriers disturbs this equilibrium state (State 2), by releasing the additional screening charges and initiating a redox reaction, it generates ROS. The reaction persists until a new state of equilibrium is attained (Cheng et al., 2021). Upon release of the applied stress, polarization is restored, resulting in the generation of additional intrinsic charges. Combined with opposite-polarity charges from the electrolyte, these charges facilitate the redox reaction (State 3). This cycle continues until the piezocatalyst reverts to its initial state. In essence, the process of accumulating and releasing screening charges enhances the piezoelectric catalytic reaction (Zhu et al., 2023). Following energy band theory, applying mechanical stress to the piezoelectric material instigates a polarization-induced piezo potential. This alteration in potential initiates a movement of band energy from the positive side to the negative side at the positive piezo potential edge (Wang et al., 2022). Consequently, the conduction band and valence band curve across the nanosystem, creating a gradient that pushes free electrons and holes away from the crystal surface. Moreover, the band bending diminishes the potential energy gap amongst the band edges and the redox potential of H_2_O/•OH and O_2_/•O_2_. This decrease enables electrons and holes to become more prone to reacting with dissolved water and oxygen, resulting in the generation of ROS (Mondal et al., 2022c). Verifying the particular reactive radical (ROS) accountable for dye degradation can be accomplished through the scavenger experiment outlined in the subsequent section (Section 3.6). The subsequent stages encompass the degradation of dye by reactive species (e^−^, h^+^, OH^−^, and O_2_^−.^) (Roy et al., 2024)

$$\text{Sample}+\text{Ultrasonication }\left(\text{US}\right)\to \text{Sample }\left(e^{-}+h^{+}\right)$$

$$e^{-}+O_{2}\;\to O_{2}^{-.}$$

$$h^{+}+H_{2}O\;\to \;.\text{ OH}$$

$$\left..\text{ OH}+\text{dye }\to \text{Degradation products}{\;(\text{CO}}_{2}{+H}_{2}O\right)$$

$$\left.O_{2}^{-.}+\text{Dye}\to \text{Degradation products}{\;(\text{CO}}_{2}{+H}_{2}O\right)$$

#### 2.3.2 ROS scavenging experiment

**FIGURE 4:**
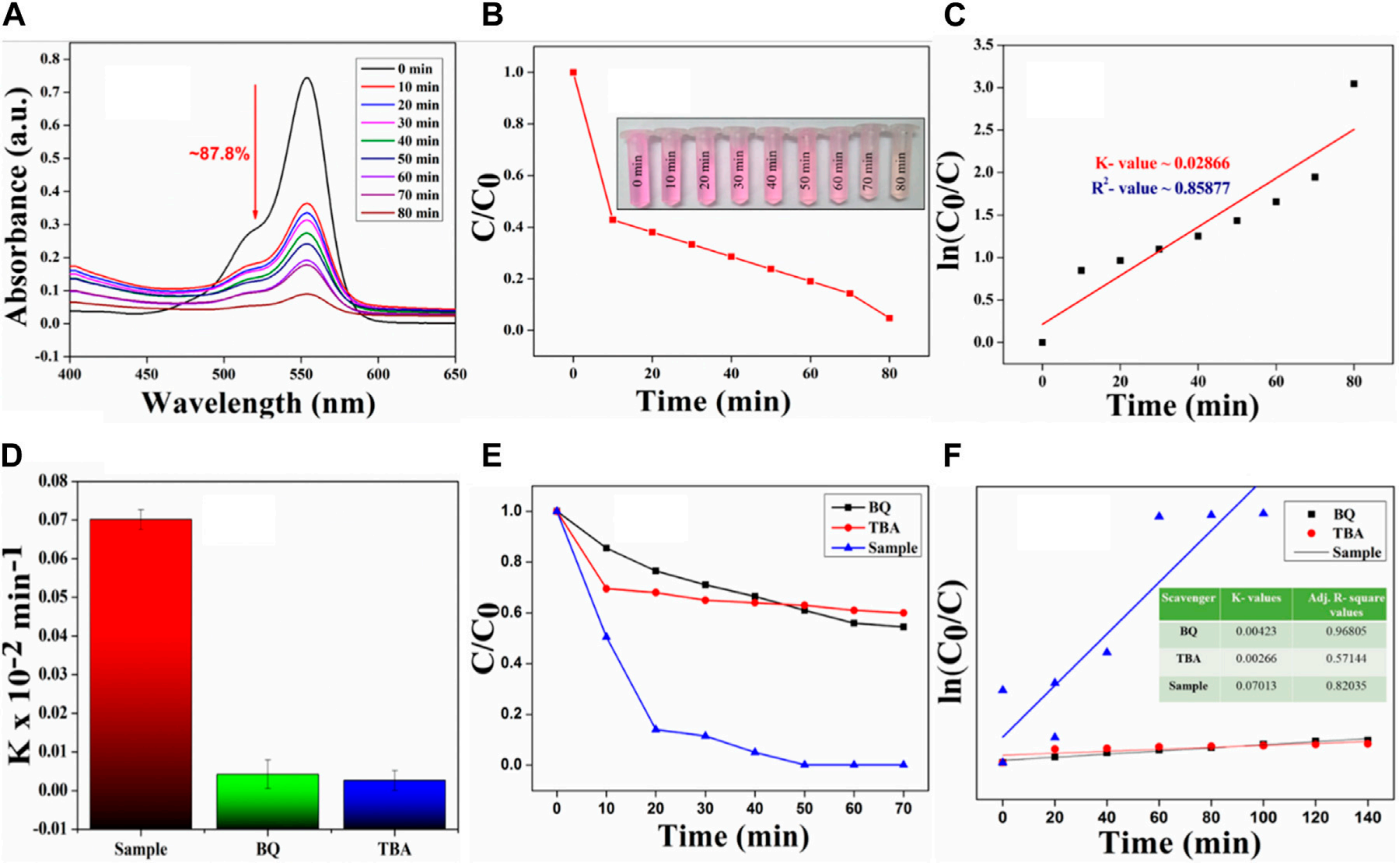
**(A)** UV-Vis spectra showing the dye degradation using the piezo catalyst of BCH 7.5 sample **(B)** C/C_0_ vs time plot shows the degradation kinetics of RhB and a real-life digital image of RhB dye degradation (Inset) **(C)** ln (C_0_/C) *versus* ultrasonic treatment time shows the kinetic rate constant value of the catalyst **(D)** Bar diagram representing the kinetic rate constants for RhB degradation were determined using samples treated with various scavengers for RhB **(E)** Scavenging experiments for dominating reactive oxygen species **(F)** First-order reaction kinetics of the catalysis experiments using various scavengers.

Performing a scavenger experiment is crucial for determining which superoxide/radical is accountable for breaking down dye molecules (Figure 4) (Lin et al., 2022). The degradation curves depicted in the figure demonstrate a decrease in catalytic degradation efficiency after the addition of scavengers, indicating their direct involvement in the RhB degradation process. Specifically, the introduction of tert-butyl alcohol (TBA) led to a reduction in reaction rate constants. The confining of hydroxyl radicals (OH•) by tert-butyl alcohol (TBA) pronouncedly reserved RhB degradation. Conversely, the addition of BQ (•O_2−_) had negligible effects on degradation percentages. Our results underscore the significant contribution of TBA, representing hydroxyl radicals, in organic dye degradation, including RhB. These ROS scavenging examinations provide valuable comprehension into the degradation appliance, highlighting the significance of specific reactive species, especially hydroxyl radicals, in the degradation procedure. The bar diagram shows a comparison between the degradation kinetics of different scavenging agents.

Notably, the ROS scavenging experiments, rapid degradation rate (*viz.* 0.02866 min^−1^), and almost 87.8% degradation of Rh-B suggest that the synthesized catalyst is capable of producing enormous amounts of ROS (especially hydroxyl radicals), which in turn degrades the dye quickly under the soft ultrasound stimulation. It is established that such kind of ROS can also be used to eradicate pathogenic bacteria. Thus, we conducted different antibacterial assays against *S. aureus* bacteria in the following section (Section 3.5) to validate our idea.

### 2.4 Antibacterial efficacy and its mode of action

**FIGURE 5:**
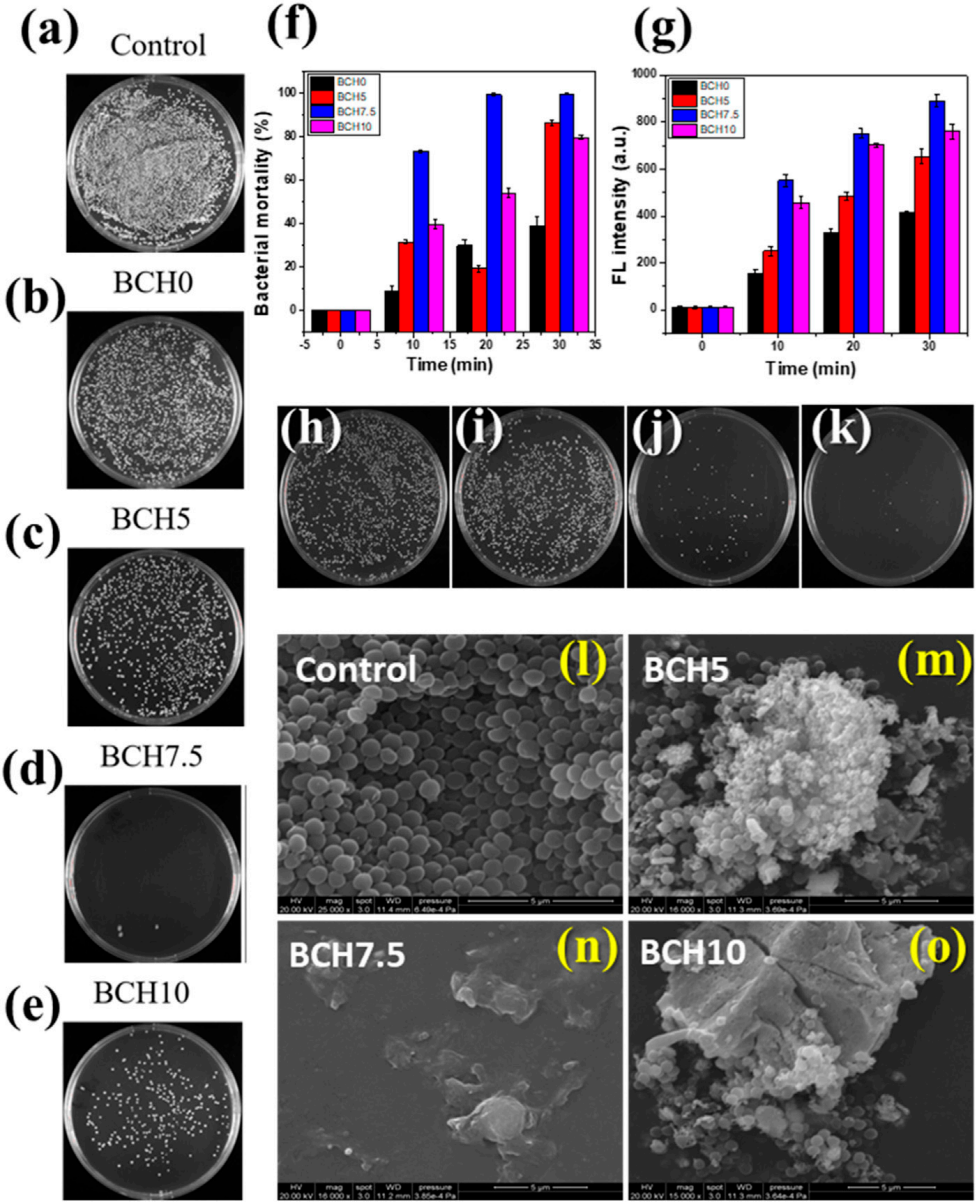
**(A–E)** Agar plate data for the different as prepared samples under US stimulus for 30 min, **(F)** bacterial mortality data from the agar plate counting, **(F, G)** Fluorescence intensity of DCF signifying ROS generation during the experiment; **(H–K)** positive and negative controls along with treated samples under the ultrasound stimulus, **(L–O)** FESEM micrographs depicting bacterial morphologies for different treated and control samples.

The effect of ultrasonic vibrations on piezocatalytic bacterial disintegration and wastewater disinfection was investigated for 30 min. A decrement in 99% colony-forming units (CFUs) on agar plates over time was significant, as depicted in Figure 5. BCH nanoparticle-treated plates under soft ultrasonically exposed cultures (b-e) exhibited marginal reductions with time, whereas a significant decrement was found for the cultures treated plates with BCH 7.5 treated nanoparticles and ultrasonication. Notably, BCH nanoparticle treatment for just 30 min of sonication led to the removal of approximately 99% of *S. aureus* bacteria, highlighting their efficacy in bacterial inactivation under mechanical stress via piezocatalysis. The time-dependent log reduction values shown in Figure 5B indicate a little decline in BCH nanoparticle-treated plates (B) that is ascribed to physical interactions, whereas mechanical stress from acoustic energy is the cause of the degradation in (C).

To study the details of the antibacterial efficacy and its mode of action, the quantification of reactive oxygen species (ROS) production was conducted. Negligible ROS generation was observed in samples BCH0, BCH5, and BCH10, while sample BCH 7.5 showed a sharp rise increment in ROS production in DCF fluorescence, indicating significant intracellular oxidant production (Figure 5F,G). This suggests that under mechanical stress, BCH 7.5 nanoparticles, induce substantial free radicals through piezocatalytic properties.

Thermal, mechanical, and chemical stress are frequently involved in the piezocatalytic elimination of harmful microorganisms using ultrasonic waves (Ali et al., 2023). Vibrations produce oxidative species such as O_2_−, H_2_O_2_, *OH, h+, and e−, which first rupture the cell wall of the bacteria, then intensify damage to the cytoplasmic membrane, and finally result in cellular material leaking that kills the bacteria (Kumar et al., 2019). Additionally, through the development of increased pressure and vibrational force, piezoelectric materials work in combination to cause cell rupture.

Piezoelectric material under mechanical stress generates high pressure, causing atomic displacement and internal polarization. The ROS generation occurs due to vibrations that induce cyclic compression and rarefaction. Positive charges generated by the piezocatalytic effect induce an electrostatic force on the negatively charged membrane of *S. aureus*. This weakens bacterial defense mechanisms, impairs metabolic processes, and disrupts electron transfer across the membrane. ROS further oxidizes lipid proteins and then induces oxidative stress on subcellular components, inhibiting proper cellular function (Ali et al., 2023). Ultrasonication-exposed BCH samples exhibit bactericidal properties associated with high ROS generation. FESEM micrographs reveal significant distortion, crumpling, and damage to bacterial cell membranes (Figure 5L–O). Interaction with BCH5 and BCH 10 nanoparticles causes folds and slight dents in the membrane, while BCH7.5 shows distinct dents and perforations. The piezocatalytic nature of BCH nanoparticles leads to irreversible damage to the cell membrane, resulting in cell death. Overall, BCH demonstrates the potential for water disinfection by eliminating coliform bacteria within 30 min of ultrasonication. Its ability to induce ROS production and disrupt bacterial membranes highlights its efficacy in bacterial inactivation, offering a promising approach for wastewater treatment.

Live/dead cell assay is used to assess the cell viability of the samples which distinguishes between live cells that interact with the cell membranes and are metabolically active, and dead cells that are not viable (Hu et al., 2022).

To demonstrate the feasibility of the proposed method, live cell cultures have been imaged and analyzed (summarized in Supplementary Figure S9). A Live/Dead BacLight bacterial viability kit was utilized for this purpose to examine the veracity of the *S. aureus* bacterial membrane and cultured with a control, BCH0, BCH 5, BCH 7.5, BCH 10 samples (1 mg mL^−1^) at 37°C for 2 h.

The suspensions were tainted with two different live/dead dye solutions. 0.5 mL and 5 µM of propidium iodide (Pl), and 1 µM SYTO nine was used for this method for 30 min. After the staining method, the cells were visualized under the microscope stage, and measured by fluorescence signals which were observed using laser scanning microscopy (ZEISS LSM 780, Oberkochen, Germany) with a ×60 coil objective lens.

The green While the red PI dye only penetrates bacteria with broken cell membranes, the SYTO 9 dye penetrates both the intact and damaged bacterial cell wall. The intensity of the green SYTO 9 dye is nearly the same for all the samples. On the other hand, the intensity of the red fluorescence is negligible for the control and BCH0 sample whereas it is continuously increasing with the increment of the doping percentages of the sample. The fluorescence is highest for the BCH7.5 sample as cells transit to a dead state. It has been confirmed from the experiment that the bacterial cell walls are ruptured maximum for the BCH7.5 sample. In addition, a merged data set is also represented in Supplementary Figure S9 where it has been seen that the red fluorescence increases with the doping percentages of the sample which validates that the cells are becoming damaged.

## 3 Conclusion

In order to eliminate the carcinogenic RhB dye and pathogenic *S. aureus* bacteria by using a facile ultrasound-based wireless approach. Herein, we developed a bismuth ferrite (BFO) doped chitosan nanocomposite through a simple solution casting method. The structural and morphological analyses of the samples were conducted by using X-ray diffraction, infrared spectroscopy, and electron microscopy confirming the successful synthesis of the sample. Further investigation reveals the piezo-responsive characteristics of the samples, which have been utilized for degrading organic dye (RhB) and pathogenic bacteria by the generation of ultrasound-mediated ROS production. Herein, we found a rapid and enormous production of ROS (especially hydroxyl radicals), which helps eradicate the carcinogenic dye and bacteria under the soft ultrasonic stimulus. The BCH nano-catalyst demonstrated exceptional degradation efficiency (∼87.8%) with a notable rate constant (k ≈ 0.02866 min^−1^), achieving nearly 88% dye degradation within 80 min and ∼99% in 30 min for bacterial death. Thus, it can be said that this nanocomposite achieved better activity compared to a single material (either BFO or chitosan). The nanocomposite shows simultaneous band edge tilting and screening charge effect, which makes it a better alternative than other piezoelectric biomaterials.

Mechanistically, the catalyst generated various reactive radicals (ROS), including hydroxyl radicals (•OH), holes (h+), superoxide radicals (•O_2_−), and electrons (e−), contributing to its high piezo-catalytic activity. Such generation of ROS has been detected by chemical scavenging experiments and further, it elucidates that hydroxyl radicals are the dominating factor of eliminating both organic dye and pathogenic bacteria. Additionally, it successfully generates 12.05 V of open circuit instantaneous piezo voltage by finger tapping, which marks a significant advancement in piezoelectric ferrite technology for environmental remediation.

In the future, there is a scope to improve the material performance by incorporating various nanomaterials (such as metal oxides, carbon-based nanomaterials, and quantum dots). Surface modification or surface functionalization can also be responsible for the enhancement of the material performance. The performance of chitosan-based materials in piezocatalysis can be significantly improved, paving the path for their practical application in environmental remediation and antibacterial treatment. However, focusing on the scalability and cost-effectiveness of the synthesis methods facilitates the large-scale production and commercialization of chitosan-based piezocatalytic materials. Shortly, these materials can be used in different biomedical devices as new and advanced composite materials are needed for this hour. Exploration of sustainable and eco-friendly synthesis routes can further enhance the attractiveness of these materials for real-world applications.

## Data Availability

The original contributions presented in the study are included in the article/Supplementary Material, further inquiries can be directed to the corresponding author.
