# Supplementary material for: Highly efficient piezocatalytic composite with chitosan biopolymeric membranes and bismuth ferrite nanoparticles for dye decomposition and pathogenic S. aureus bacteria killing
Source: Front Chem. 2024 Jun 6;12:1420040. doi: 10.3389/fchem.2024.1420040 (PMC11187321; doi:10.3389/fchem.2024.1420040)
Supplement: Supplementary file 1 [file DataSheet1.docx]

**Highly Efficient Piezocatalytic Composite with Chitosan Biopolymeric Membranes and Bismuth Ferrite Nanoparticles for Dye Decomposition and Pathogenic *S. aureus* Bacteria Killing**

Yunhong Liu,^1^ Jhilik Roy^2^, Shubham Roy^3^, Nur Amin Hoque^4^, Bing Guo^3*^

^1^ Department of Clinical Lab, The People's Hospital of Longhua, Shenzhen, 518109, China

^2^ Department of Physics, Jadavpur University, Kolkata-700032, India

^3^ School of Science, Shenzhen Key Laboratory of Flexible Printed Electronics Technology, Shenzhen Key Laboratory of Advanced Functional Carbon Materials Research and Comprehensive Application, Harbin Institute of Technology, Shenzhen-518055, China

^4^ SAIS Department, Indian Association for the Cultivation of Science, Kolkata, 700032, India

Corresponding author: B. Guo (guobing2020@hit.edu.cn)

**Supporting information**

**S1. Materials and method**

**S1.1 Materials**

From Merck, India, acetone (HPLC grade), ethanol (HPLC grade), bismuth (III) nitrate (Bi (NO_3_)_3_·5H_2_O), and iron (III) nitrate (Fe (NO_3_)_3_.9H_2_O) were obtained. From Sigma Aldrich, ethylene glycol, citric acid, and 2-methoxyethanol were obtained. Without any additional purification, all of the analytic-grade reagents were employed. For every experiment, Millipore water with a resistivity of 18.2 MΩ-cm was utilized. Sigma-Aldrich provided the p-benzoquinone (BQ) and chitosan powder. Dimethylformamide (DMF) (HPLC grade) and rhodamine B (RhB) were acquired from Merck, India, and Loba Chemie Pvt. Ltd., respectively.

**S1.2** **Synthesis of the piezocatalytic bismuth ferrite (BFO) NP_S_**

Bismuth ferrite powder was synthesized via the solvothermal synthesis method. Bi (NO_3_)_3_·5H_2_O, Fe (NO_3_)_3_.9H_2_O (0.002 mol each) were dissolved in 2-methoxy-ethanol (15 ml). After 40 min of stirring, 0.002 mol citric acid and 0.002 mol ethylene glycol were added and then again stirred for 60 min at 60°C. The prepared solution was further transferred to a Teflon-lined stainless-steel autoclave which was heated at 100°C for 16 h. lastly, the precipitates were collected, washed several times, and dried at 70°C. The powder was then calcinated at 500°C for 2 h, and then ground to obtain BFO.

**S1.3 Synthesis of piezoelectric** **BFO-incorporated chitosan (BCH) nanocomposites**

BFO-doped chitosan nanoparticles were synthesized via a solution casting synthesis method (Fig. 1A). At first 0.5 gm of chitosan powder was added to the 50 ml 1M acetic acid and, a homogeneous gel-like solution was formed. Then it was heated at 50°C and was stirred for 3 hrs. Then BFO powder in three different percentages (5%, 7.5%, 10%) was further added to the mixture and was stirred overnight. The gel-like nanocomposites were then cast into clean Petri dishes and dried at 50 °C in a hot air oven for 6 hours to obtain flexible, free-standing membranes marked as BCH5, BCH7.5, and BCH10. A pristine chitosan film was also prepared using the same procedure without nanoparticle addition, labeled as BCH0 for comparison.

**S1.4 Experimental Characterization Techniques of BFO-doped Chitosan (BCH) Membranes**

The structural parameters and phase purities of the synthesized BCH membranes were investigated through an X-ray diffraction technique within the angle of 20⁰ to 80⁰ (2θ). The diffractometer was equipped (Bruker AXS in Wisconsin, USA (Cu-Kα target- 1.54 Å)) with a power of 35 kV and 35 mA.

The vibrational spectroscopy and information related to the bonding networks of the samples were investigated by Fourier transform infrared (FTIR) spectroscopy within the range of 400 to 4000 cm^−1^.[28] The fabricated thin biopolymeric films were placed in the path of the IR source to find the transmission spectra.

The transmission electron microscope was used to investigate the structural and morphological properties of synthesized bismuth ferrite NPs operating at 200 kV (JEM-2100 Plus). Further, the surface morphologies of the membranes were investigated by employing an Inspect-F50, FEI field-emission scanning electron microscope (FESEM) operating at 20 kV having a chamber pressure of 3x10^-4^ Pa. The elemental compositions were investigated by a Bruker EDAX attachment in build with FESEM instrument.

The thermal stability of the synthesized membranes was investigated by thermogravimetric (TGA) analysis using a DTG-60H, Shimadzu instrument in a nitrogen atmosphere.

The piezoelectric performance of the nanocomposite membrane was investigated by a digital storage oscilloscope (DSO: Keysight technologies) operating at a fixed bias voltage of 250 mV

The organic dye degradation was investigated through a UV spectrophotometer (Lambda-25 r from Perkin Elmer) within the wavelength range of 400-800 nm.

**S2. Antibacterial experiments**

To assess the efficacy of BCH nanocomposites in piezocatalytic bacterial disinfection under ultrasonic conditions, pathogenic bacteria S. aureus was used. S. aureus cells (ATCC 29213) were cultured overnight in Mueller Hinton Broth (M-H Broth) and then inoculated into sterile broth followed by the incubation process at 37 °C until it reached 0.5 McFarland standard. The culture was separated into four parts: firstly, control, then with BCH0 nanoparticles, with BCH5 nanoparticles, and lastly with BCH7.5 nanoparticles, with BCH10 nanoparticles. After 30 minutes of ultrasound treatment, the aliquots were collected and diluted in sterile PBS and smeared on MH-agar plates. After 24 h of incubation at 37 °C, the colony counting method was adopted to calculate the bacterial mortality values employing the following equation.[48]

$$Bacterial mortality (\%)=(\frac{N_{0}}{N}x 100)$$

Where N_0_ denotes the CFU/mL counted from the *S. aureus* bacterial colony at t=0, and N denotes the bacterial colony at t=30 min.

Bacterial FESEM was conducted to examine the bacterial morphology and membrane continuity. Samples from every group were collected and rinsed with filtered PBS solution (5 minutes each at 5000 rpm) twice. Subsequently, 2 h fixation was performed by adding 100 μL of 2.5% glutaraldehyde in every nanocomposite. After that drop-casting method was employed to find the bacterial samples for FESEM measurement by serial dilution using ethanol.

A conventional DCFDA technique has been adapted for ROS generation. In this context, 1 ml bacteria containing aqueous solutions were collected every 15 min during the experiments and centrifuged and washed with PBS buffer solution followed by the addition of 1 ml for the estimation of reactive oxidative species (ROS) generation, 1 mL, 10 µM DCFDA solution. And collected the fluorescence intensity using a fluorescence spectrophotometer (BIOTEK) at the wavelength of 529 nm (excitation wavelength 504 nm).

**S3. Piezocatalytic experiments**

First, 20 mL of dye solutions were produced in a glass container. After that, 1 mg/mL (w/v) of the piezocatalyst was added to the container. The solutions were subjected to 10-minute intervals of ultrasonic vibration. At a wavelength of 554 nm, a portion of the solutions were taken out of the assigned vials, and their optical density was calculated. These experiments suggest the promising piezocatalytic degradation of carcinogenic organic dye and its underlying mechanisms.

Additionally, the piezo-catalysis experiment was performed at a constant temperature of approximately 25 °C. To maintain this temperature, ice cubes were added to the ultrasonicator during the catalysis process. Fig. S8 shows the time-temperature curve.

The absorbance spectra illustrate the degradation kinetics of piezocatalyst (BCH 7.5). The percentage piezodynamic degradation efficiency (η) of the nanocomposite was determined by applying the following equation, [41]

Ƞ= $\frac{C_{0-}C}{C_{0}}x100 \%$ …………….. (1)

Here, C_0_ represents the starting concentration of the dye solution, while C stands for the concentration at a particular point in time.

The following relation was used to find the first-order kinetic constant (k) for each case. The estimated K values were derived from the slopes of the graph, which were generated by analyzing the natural logarithm of the ratio C/C_0_ versus time (t).[41]

$C= C_{0}exp(-kt)$……………… (2)





**Fig S1:** The FTIR analysis of the pure Bismuth ferrite (BFO) nanoparticles


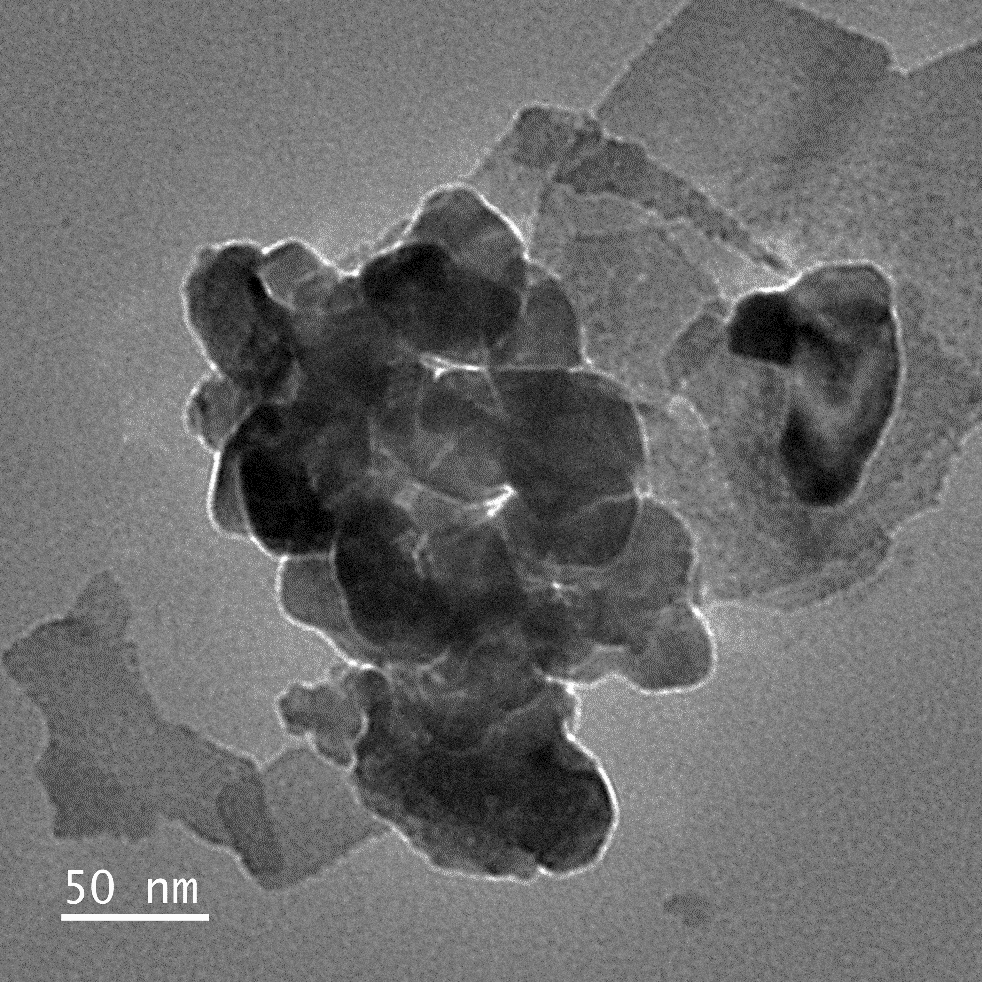


**Fig S2:** Transmission electron microscopy (TEM) images of bismuth ferrite (BFO) nanoparticles

**S4. Calculation of force**

The amount of force imparted on the membrane is estimated by the laws of conservation of energy.

mgh = ½ mv^2^…………………….(1)

Where m is the mass of a striking object, v is velocity, h is the height of the object from where it falls, and g is the acceleration due to gravity.

Velocity has been calculated by equation-1 for the height of 15cm and found to be 1.714 m/s.

Now, the equation of momentum is:

mv = (F-mg) Δt……………………..(2)

Therefore, the applied force F is:

F = m (v/Δt + g)…………………….(3)

where Δt is the full width at half maxima of the output voltage vs the time graph [S4, S5].





**Fig. S4:** Full width at half maxima (Δt) from the time vs voltage plot of the device

The output piezoelectric voltage has been estimated by exerting force by hand where m is 0.096 kg, v is 1.727 ms^-1^, h is 0.16 m, g is 9.8 m/s^2^, and Δt is 0.00287 s. Putting these values into relation 3, the applied force on the device is found to be 58.7 N.





**Fig S5:** Output voltage of BCH 7.5 nanocomposite film for the force imparting by finger tapping





**Fig S6:** UV-Vis spectra showing RhB dye degradation experimental result for control samples





**Fig S7**: The reusability test of the catalyst (BCH 7.5)





**Fig S8:** Time vs. Temperature study during the piezo catalysis using BCH catalyst


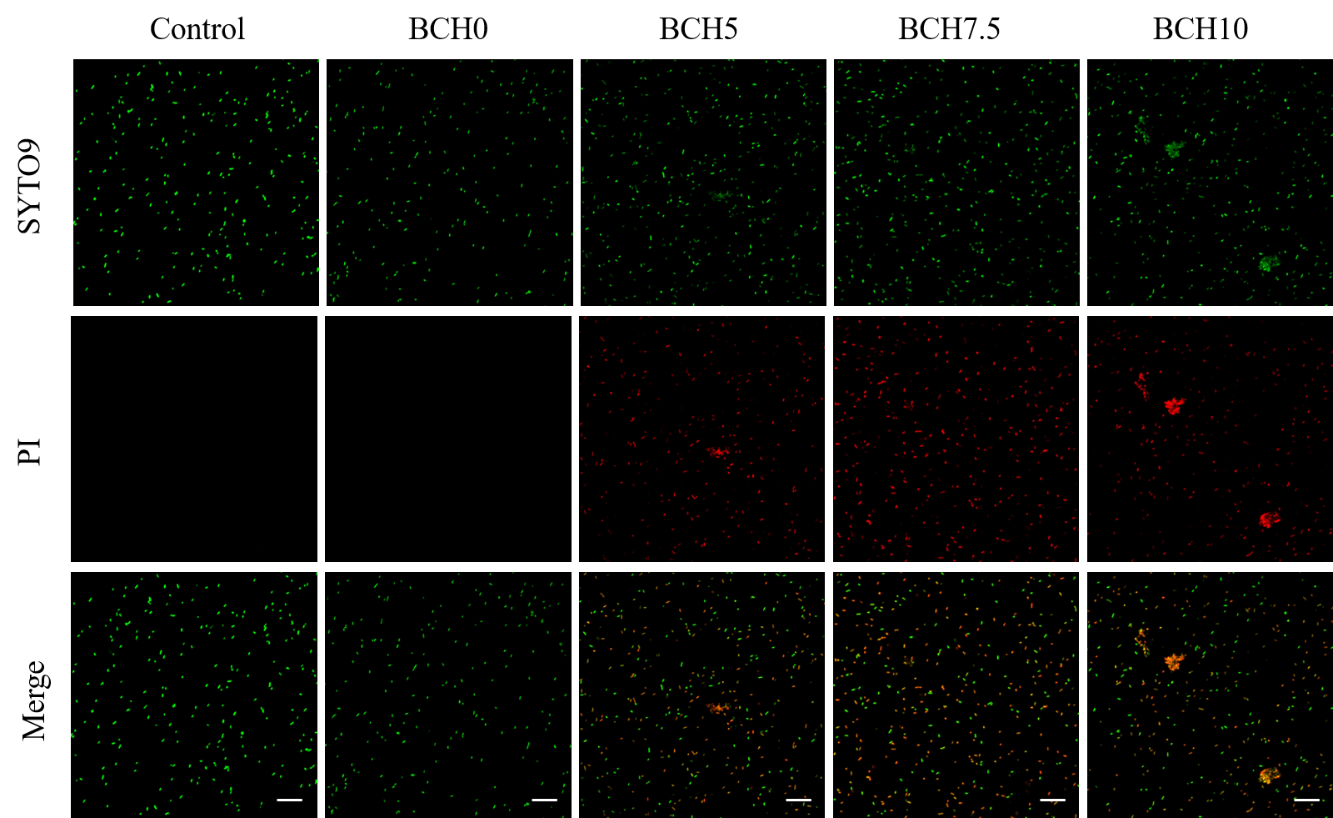


**Fig S9.** Live dead cell assay of the bacteria for different samples
